# Association between household size and COVID-19: A UK Biobank observational study

**DOI:** 10.1177/01410768211073923

**Published:** 2022-02-04

**Authors:** Clare L Gillies, Alex V Rowlands, Cameron Razieh, Vahé Nafilyan, Yogini Chudasama, Nazrul Islam, Francesco Zaccardi, Daniel Ayoubkhani, Claire Lawson, Melanie J Davies, Tom Yates, Kamlesh Khunti

**Affiliations:** 1 Leicester Real World Evidence Unit, Diabetes Research Centre, Leicester, LE5 4PW, UK; 2 Diabetes Research Centre, Leicester Diabetes Centre, Leicester General Hospital, Leicester, LE5 4PW, UK; 3 NIHR Applied Research Collaboration – East Midlands (ARC-EM), Leicester General Hospital, Leicester, LE5 4PW, UK; 4 National Institute for Health Research (NIHR), Leicester Biomedical Research Centre (BRC), Leicester General Hospital, Leicester, LE5 4PW, UK; 5 Office for National Statistics, Government Buildings, Newport, South Wales, NP10 8XG, UK; 6 Nuffield Department of Population Health, University of Oxford, Oxford, OX1 2JD, UK

**Keywords:** Infectious diseases, epidemiologic studies, housing and health, public health, social conditions and disease

## Abstract

**Objective:**

To assess the association between household size and risk of non-severe or severe COVID-19.

**Design:**

A longitudinal observational study.

**Setting:**

This study utilised UK Biobank linked to national SARS-CoV-2 laboratory test data.

**Participants:**

401,910 individuals with available data on household size in UK Biobank.

**Main outcome measures:**

Household size was categorised as single occupancy, two-person households and households of three or more. Severe COVID-19 was defined as a positive SARS-CoV-2 test on hospital admission or death with COVID-19 recorded as the underlying cause; and non-severe COVID-19 as a positive test from a community setting. Logistic regression models were fitted to assess associations, adjusting for potential confounders.

**Results:**

Of 401,910 individuals, 3612 (1%) were identified as having suffered from a severe COVID-19 infection and 11,264 (2.8%) from a non-severe infection, between 16 March 2020 and 16 March 2021. Overall, the odds of severe COVID-19 was significantly higher among individuals living alone (adjusted odds ratio: 1.24 [95% confidence interval: 1.14 to 1.36], or living in a household of three or more individuals (adjusted odds ratio: 1.28 [1.17 to 1.39], when compared to individuals living in a household of two. For non-severe COVID-19 infection, individuals living in a single-occupancy household had lower odds compared to those living in a household of two (adjusted odds ratio: 0.88 [0.82 to 0.93].

**Conclusions:**

Odds of severe or non-severe COVID-19 infection were associated with household size. Increasing understanding of why certain households are more at risk is important for limiting spread of the infection.

## Introduction

During 2020, COVID-19 spread rapidly across the globe: as of 5 May 2021, over 165 million cases were reported worldwide, resulting in over 3.4 million deaths. By country, the United Kingdom has had the seventh highest number of cases, totalling over 4.4 million.^1^ The severity of COVID-19 varies substantially between individuals; therefore, understanding risk factors associated with poor outcomes is an important step for identifying those most at risk from the disease. To date, research has shown a number of factors associated with poor outcomes, including ethnicity, obesity, presence of morbidity (such as type 2 diabetes), sex, and age.^2–4^ Much of the research to date has utilised hospital admissions datasets that frequently lack data on socio demographic risk factors. Therefore, rigorous evidence on the association between these factors and COVID-19 outcomes is more limited.

In the United Kingdom, a large database established in 2006 that recorded a wide range of lifestyle and socio demographic information from participants (UK Biobank) has been linked to SARS-CoV-2 laboratory test data. Here, we aim to use the UK Biobank database to assess how household size is associated with severity of COVID-19 disease.

## Materials and methods

This study is reported following the Strengthening the Reporting of Observational Studies in Epidemiology guidelines (checklist included in supplementary material, Figure S1).^5^

### Study population

This analysis was carried out using UK Biobank (application 36371), a national database containing half a million adults aged 40–69 years at study entry.^6^ Baseline measures were collected between 2006 and 2010 via interviews at one of 22 UK Biobank centres. UK Biobank data have been linked to national SARS-CoV-2 laboratory test data through Public Health England’s Second Generation Surveillance System.^7^ For this analysis, linked data on COVID-19 outcomes were available from 16 March 2020 to 16 March 2021.^7^ As COVID-19 testing data were only available for England, participants from non-English centres were removed from the cohort, as were those who died before 16 March 2020, as this was the first COVID-19 testing date.

### Exposure, outcome and covariates

Our primary exposure of interest for this analysis was household composition, as recorded at study entry. All participants filled in a baseline questionnaire, with the question on household composition asking ‘*Including yourself, how many people are living together in your household? (Include those who usually live in the house such as students living away from home during term, partners in the armed forces or professions such as pilots)*’. For this analysis, the answers were combined into three categories: single-occupancy households, households of two individuals (the reference category) and households of three or more. A household of two individuals was chosen as the reference category, as this was the largest group.^8^ The outcomes of interest were severity of COVID-19. Severe COVID-19 was defined as a positive hospital test or a death related to the disease (any death with an ICD-10 code of U07.1 or U07.2 as the underlying cause of death on the death certificate). Non-severe COVID-19 was defined as a positive test in an outpatient setting. Both severe and non-severe cases were compared against no COVID-19 (those who were not tested or who tested negative in either setting).

Covariates included in the analysis were selected based on current knowledge of potential confounders associated with COVID-19 outcomes. Patient characteristics considered were: age at time of COVID-19 test; ethnicity (classified as White European, South Asian and Black Caribbean); body mass index; deprivation (based on the Townsend score, which is a measure of material deprivation within a population based on unemployment, car and home ownership and household overcrowding); smoking status (classified as yes [current or previous] or no); sex (male or female); health worker status; current or previous cancer (self-reported; yes/no); and morbidity (classified as yes if the individual reported having one or more of the following conditions: cardiovascular, respiratory, renal, neurological, musculoskeletal, haematological, gynaecological, immunological or infections). All patient characteristics were collected at the baseline assessment carried out at study entry.^9^

### Statistical analysis

A complete case analysis fitting logistic regression models was used to compare odds of severe COVID-19 by household size, using a household of two as the reference category. All analyses were adjusted for age, sex, body mass index, deprivation, previous or current cancer, presence of morbidities, health worker status, smoking status and Townsend score. Analyses were carried out overall and then stratified by ethnicity and sex to assess if the effect of household size differed between groups. Interactions between household size and either ethnicity or sex were assessed by fitting interaction terms, and comparing model fit with and without the terms using likelihood ratio tests. All analyses were carried out in Stata 15.

## Results

After participants from Scotland and Wales were removed (n = 56,649), as well as those who died before testing for SARS-CoV-2 began (n = 25,324), 420,564 participants remained. Of these, 18,654 had missing data for covariates required for the analysis (such as household size and ethnicity): the final analysis cohort therefore comprised 401,910 individuals (80% of the starting cohort) (Supplementary Figure S2). Of those with missing data, a slightly higher percentage were classified as severe or non-severe COVID-19 compared to the analysis cohort (Table S2).

Baseline characteristics of this cohort are given in Table 1: 72,087 (17.9%) lived alone, 189,109 (47.1%) lived in a two-person household and 140,714 (35.0%) lived in a household of three or more people including themselves; 3612 (0.9%) suffered from a severe and 11,264 (2.8%) from a non-severe COVID-19 infection between 16 March 2020 and 16 March 2021. Severe COVID-19 was most prevalent in one-person households (1.2% of all two-person households, compared to 0.9% of two-person and 0.8% of households of three or more), whereas non-severe COVID-19 infection was more prevalent in larger households (4.2% of households containing three or more individuals compared to 2.1% and 2% of one- and two- person households respectively). Individuals living in households, of three or more were generally younger, were less likely to have a morbidity, such as cardiovascular, respiratory or renal disease, were more likely to be men, and less likely to be White, when compared to smaller households.

**Table 1.** Participant characteristics.

	All (N = 401,910)	Household size		
		1 person (N = 72,087)	2 persons (189,109)	3 or more (140,714)
Age at test (years)	68.2 (8.07)	68.6 (7.95)	70.58 (7.02)	62.7 (7.20)
Body mass index (kg/m^2^)	27.4 (4.74)	27.5 (5.12)	27.41 (4.60)	27.2 (4.71)
Townsend score	−1.41 (3.00)	−0.12 (3.36)	−1.79 (2.78)	−1.57 (2.90)
Sex				
Male	180,150 (44.8)	29,601 (41.1)	83,802 (44.3)	66,747 (47.4)
Female	221,760 (55.2)	42,486 (58.9)	105,307 (55.7)	73, 967 (52.6)
Ethnicity				
White European	387,771 (96.5)	69,739 (96.7)	186,006 (98.4)	132,026 (93.8)
South Asian	6959 (1.7)	596 (0.8)	1404 (0.7)	4959 (3.5)
Black and African Caribbean	7180 (1.8)	1752 (2.4)	1699 (0.9)	3729 (2.7)
Smoking status				
Never	223,207 (55.5)	37,595 (52.2)	101,171 (53.5)	84,441 (60.0)
Previous	139,625 (34.7)	24,207 (33.6)	72,544 (38.4)	42,874 (30.5)
Current	39,078 (9.7)	10,285 (14.3)	15,594 (8.1)	13,399 (9.5)
Morbidities				
Yes	299,728 (74.6)	56,300 (78.1)	147,734 (78.1)	95,694 (68.0)
No	102,182 (25.4)	15,787 (21.9)	41,375 (21.9)	45,020 (32.0)
Past or current cancer				
Yes	31,381 (7.8)	6323 (8.8)	17,266 (9.1)	7792 (5.5)
No	370,529 (92.2)	65,764 (91.2)	171,843 (90.9)	132,922 (94.5)
Health worker				
Yes	4358 (1.1)	573 (0.8)	1461 (0.8)	2324 (1.7)
No	397,552 (98.9)	71,514 (99.2)	187,648 (99.2)	138,390 (98.4)
COVID-19 status				
None	387,034 (96.3)	69,724 (96.7)	183,666 (97.1)	133,644 (95.0)
Non-severe	11,264 (2.8)	1,515 (2.1)	3,841 (2.0)	5,908 (4.2)
Severe	3612 (0.9)	848 (1.2)	1692 (0.9)	1162 (0.8)

Values reported are N (%) unless otherwise stated.

**Figure 1.**
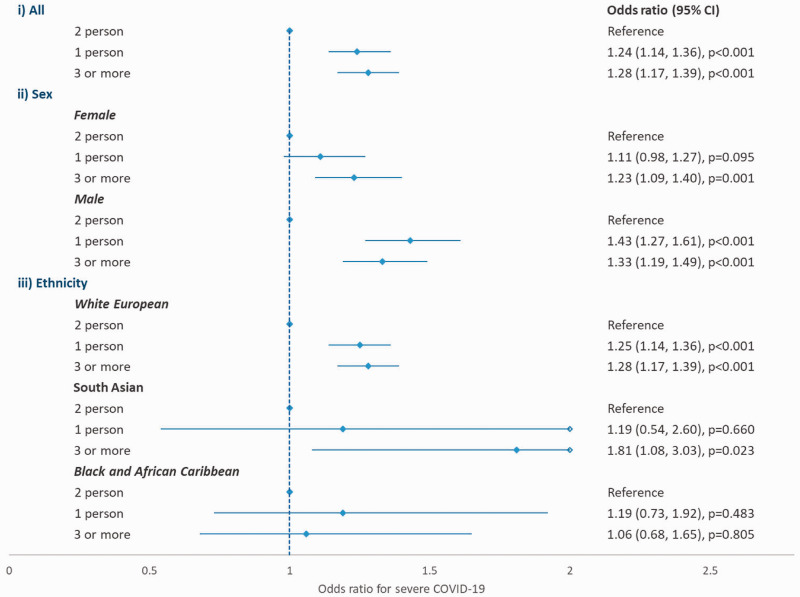
Association between household size and odds of severe COVID-19, stratified by sex and ethnicity.

Adjusting for age, body mass index, Townsend deprivation score, sex, ethnicity, morbidity, smoking status, previous or current cancer and health worker status, overall the odds of suffering from severe COVID-19 were greater among individuals living alone (adjusted odds ratio: 1.24; 95% confidence interval [CI]: 1.14 to 1.36) or living in a household of three or more individuals (adjusted odds ratio: 1.28; 95% CI: 1.17 to 1.39) when compared to individuals living in a household of two individuals (Figure 1). Stratified analysis by sex showed a potentially stronger impact of household size in men, particular for those living alone: adjusted odds ratio was 1.43 (95% CI: 1.27 to 1.61) in men and 1.11 (95% CI: 0.98 to 1.27) in women, and adding an interaction term between sex and household size was found to be statistically significant (*p* = 0.018). No statistically significant interaction was found between ethnicity and household size (*p* = 0.198).

**Figure 2.**
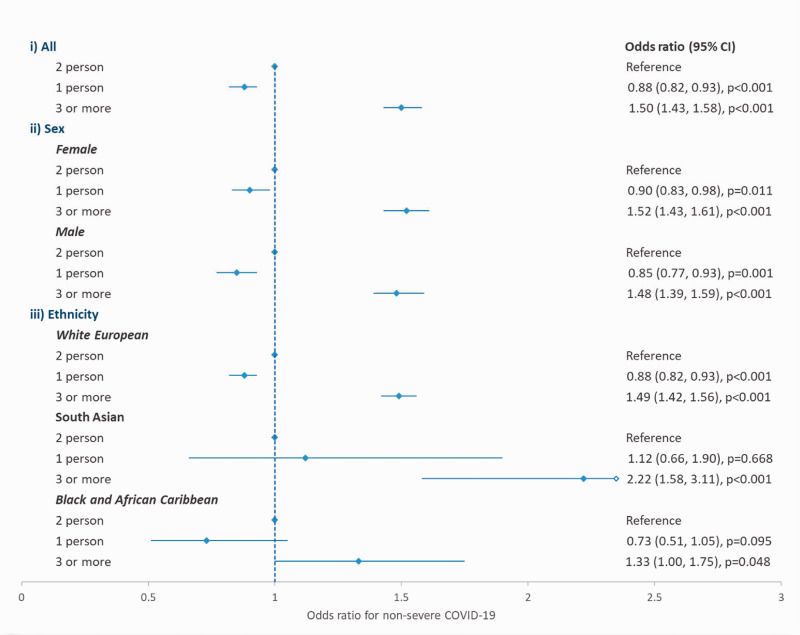
Association between household size and odds of non-severe COVID-19, stratified by sex and ethnicity.

For non-severe COVID-19 infection (Figure 2), the odds were reduced in single-occupancy households compared to households of two people (adjusted odds ratio: 0.88; 95% CI: 0.82 to 0.93), but increased in those of three or more (adjusted odds ratio: 1.50; 95% CI: 1.43 to 1.58). The same pattern of odds was seen when the analyses were stratified by sex and ethnicity, and fitting interaction terms between household size and either sex or ethnicity did not significantly improve model fit (*p* = 0.2081 and *p* = 0.2063, respectively). Full results for both the logistic regression models fitted are given in the supplementary material (Table S1).

## Discussion

In this study, household size has been shown to be an important risk factor for both severe and non-severe COVID-19, even after adjustment for potential confounders such as deprivation, prior morbidity and age. This may be because, as has been shown in previous research, older people living alone were more likely to have received help from carers and informal helpers during the pandemic lockdown periods, when compared to individuals living with at least one other adult.^10^ As such, adults in single-occupancy households were more likely to be exposed to frequent contacts with people from different households. Individuals living alone are also more likely to present at hospital and to be admitted, as they have no support network or care from within their household. As shown in previous research, which reported that adults over the age of 65 who lived alone were more likely to utilise a hospital emergency department (odds ratio: 1.50; 95% CI: 1.16 to 1.93), and to be admitted to hospital (odds ratio: 1.30; 95% CI: 0.99 to 1.70) than those living with someone else.^11^ Our research also showed that the impact of household size differed by sex for severe COVID-19, with men at greater odds than women if they lived alone; this may be because during the pandemic, reliance on carers and accessing healthcare differed by sex, but we have found no published evidence to support this. Individuals in larger households, being at increased odds of non-severe COVID-19, may be due to increased mixing of these households. Households of three or more are more likely to include individuals of working age, or children, than households of just one or two people. This will increase their external exposure to COVID-19 from outside the household.

Research to date has found associations between household size and COVID-19 infection and outcomes, but with household size analysed in different ways it is difficult to compare results across studies. A study assessing average household size and incidence rates of COVID-19 in New York City found that average household size was the single most important driver in variation in COVID-19 incidence rates, explaining 62% of variation, while population density by itself was not significantly associated with incidence.^12^ A study using UK census records linked to hospital episodes data found that living in a multi-generational household, even when dependent children were not part of the household, was associated with an increased risk of COVID-19 death.^9^ Such increased risk was found to explain around 11% of the higher risk of COVID-19 death among older women from a South Asian background but very little for South Asian men, or people in other ethnic minority groups. A further study, analysing the association between SARS-CoV-2 PCR positivity and household size as a continuous variable, estimated an adjusted odds ratio of 1.06 (95% CI: 1.02 to 1.11) for PCR positivity with increasing household size.^13^ Therefore, although previous research has shown an association between household size and COVID-19, by analysing household size as a categorical variable in this study we have been able to determine the households with the highest risks.

The UK Biobank dataset provides the opportunity to explore risk factors not routinely collected in other datasets. It is a large cohort of over half a million participants, contains in-depth health information, and is regularly augmented with additional data, such as the COVID-19 testing datasets utilised here. UK Biobank does have limitations though; in particular, baseline data were collected at study entry in 2011, and individual’s circumstances may have changed since then. Given we would expect changes in living circumstances to be random, the impact of misclassification of household size will be to dilute associations,^14^ and the results presented here are likely to be an underestimation of the true effects. In addition, household size is not commonly available in other datasets, so the UK Biobank dataset was the best option to address this research question, despite the limitations of the data. Also, although disease severity of COVID-19 was consistent with the definition proposed by the researchers who developed the linkage between Biobank and COVID-19 datasets,^7^ those classified as non-severe because they have a positive outpatient test, may have gone on to be hospitalised at a later date. In addition, some individuals with non-severe COVID-19 may have chosen not to undertake a test; they therefore would be misclassified as non-COVID-19 in this analysis. If misclassification of COVID-19 status was associated with cohort characteristics such as age, sex and ethnicity, this could have potentially affected our results. Furthermore, UK Biobank is not completely representative of a UK population with participants generally older, more likely to be women, and to live in less socioeconomically deprived areas than non-participants; and less likely to be obese or smoke and with fewer self-reported health conditions than the general population.^15^ Nonetheless, valid assessment of exposure-disease relationships do not require participants to be fully representative of the population at large.^16^ A further limitation is that at the start of follow-up in this study, testing for COVID-19 in the UK was targeted, at least during the early stage of the pandemic, meaning the cohort analysed in this study may be prone to biases.

## Conclusions

In conclusion, living in a household of two people is associated with lower odds of severe COVID-19 compared to living alone or in a household of three or more, after adjustment for potential confounding factors. It was also found that living alone is a greater risk factor in men than in women. For non-severe COVID-19 infection, the lowest odds were found in individuals living alone. Understanding risk factors associated with COVID-19 transmission and severity is important, as this will influence advice and policies surrounding future waves of COVID-19 as well as future infectious disease epidemics.

## Supplemental Material

sj-pdf-1-jrs-10.1177_01410768211073923 - Supplemental material for Association between household size and COVID-19: A UK Biobank observational study Click here for additional data file. Supplemental material, sj-pdf-1-jrs-10.1177_01410768211073923 for Association between household size and COVID-19: A UK Biobank observational study by Clare L Gillies, Alex V Rowlands, Cameron Razieh, Vahé Nafilyan, Yogini Chudasama, Nazrul Islam, Francesco Zaccardi, Daniel Ayoubkhani, Claire Lawson, Melanie J Davies, Tom Yates and Kamlesh Khunti in Journal of the Royal Society of Medicine

## Declarations

### Guarantor

CG
